# Case Report: A Pathogenic Missense Variant of *WT1* Cosegregates With Proteinuria in a Six-Generation Chinese Family With IgA Nephropathy

**DOI:** 10.3389/fmed.2021.810940

**Published:** 2022-01-31

**Authors:** Qianqian Li, Li Zhu, Sufang Shi, Damin Xu, Jicheng Lv, Hong Zhang

**Affiliations:** Renal Division, Department of Medicine, Peking University First Hospital; Peking University Institute of Nephrology; Key Laboratory of Renal Disease (Peking University), National Health Commission; Key Laboratory of Chronic Kidney Disease Prevention and Treatment, Ministry of Education, Beijing, China

**Keywords:** IgA nephropathy, proteinuria, *WT1* gene, *NPHS1* gene, pedigree

## Abstract

Immunoglobulin A (IgA) nephropathy (IgAN) is the most common type of primary glomerulonephritis worldwide. In addition to hematuria, proteinuria is observed in a considerable proportion of patients with IgAN and has proven to be a strong risk factor for disease progression. Although the exact pathogenesis of IgAN is still unclear, genetic factors are widely considered to play a role in its occurrence and development. Here, we investigated a large IgAN-associated pedigree of 47 members belonging to six generations. Two members of the family who presented with proteinuria and hematuria were diagnosed with IgAN through renal biopsy. Four other members also exhibited proteinuria or hematuria but without renal biopsy. Using whole-exome sequencing, we identified a likely pathogenic variant in *WT1* (c.1397C>T; p.Ser466Phe) that cosegregated with proteinuria in the affected family members. In addition, another pathogenic variant in *NPHS1* (c.3478C>T; p.Arg1160Ter) was identified; however, it did not cosegregate with abnormal proteinuria. Compared to individuals in the pedigree with only one heterozygous *WT1* variant (c.1397C>T; p.Ser466Phe), the proband and her younger brother carried an additional *WT1* variant (c.1433-10G>A) and presented with a more severe phenotype and rapid progression to end-stage kidney disease. Our findings suggest the *WT1* missense variant (c.1397C>T; p.Ser466Phe)-induced primary podocyte injury might contribute to the proteinuria phenotype and IgAN progression in this pedigree.

## Introduction

Immunoglobulin A (IgA) nephropathy (IgAN) is the most common primary glomerulonephritis worldwide and has complex and unclear pathogenesis. IgAN can occur as a sporadic or familial disease depending on the clinical characteristics of the disease. Compared to sporadic IgAN cases, familial IgAN cases have earlier onset and poorer renal outcomes (1). Numerous familial IgAN reports have shown that genetic factors are involved in its occurrence and development (2, 3). Although hematuria is the most common clinical manifestation of IgAN, proteinuria is a more widely known risk factor for progression to end-stage kidney disease (ESKD) than hematuria in patients with IgAN (4).

The Wilms tumor suppressor 1 (*WT1*) gene, located on chromosome 11p13, contains 10 exons and encodes a transcription factor of the zinc finger protein family. This transcription factor plays a crucial role in the development of the kidney and genitals (5). The *WT1* gene is predominantly expressed in maturing podocytes in adult kidneys, and is related to the glomerular filtration barrier function, especially in proteinuria (6). Previous studies have reported that mutations in the *WT1* gene are related to Frasier syndrome, Denys-Drash syndrome (DDS), focal segmental glomerulosclerosis (FSGS), and nephrotic syndrome, indicating that *WT1* might play an essential role in the differentiation and function of podocytes (6, 7).

Genetic sequencing in familial IgAN cases can help identify causal genes of IgAN and may facilitate the elucidation of the mechanism of IgAN development and progression. In this case study, we report a pathogenic missense variant in the *WT1* gene in a large pedigree. Two members presented with biopsy-proven IgAN, which suggests that the *WT1* gene may be related to the pathogenesis of IgAN.

## Case Presentation

**Figure 1:**
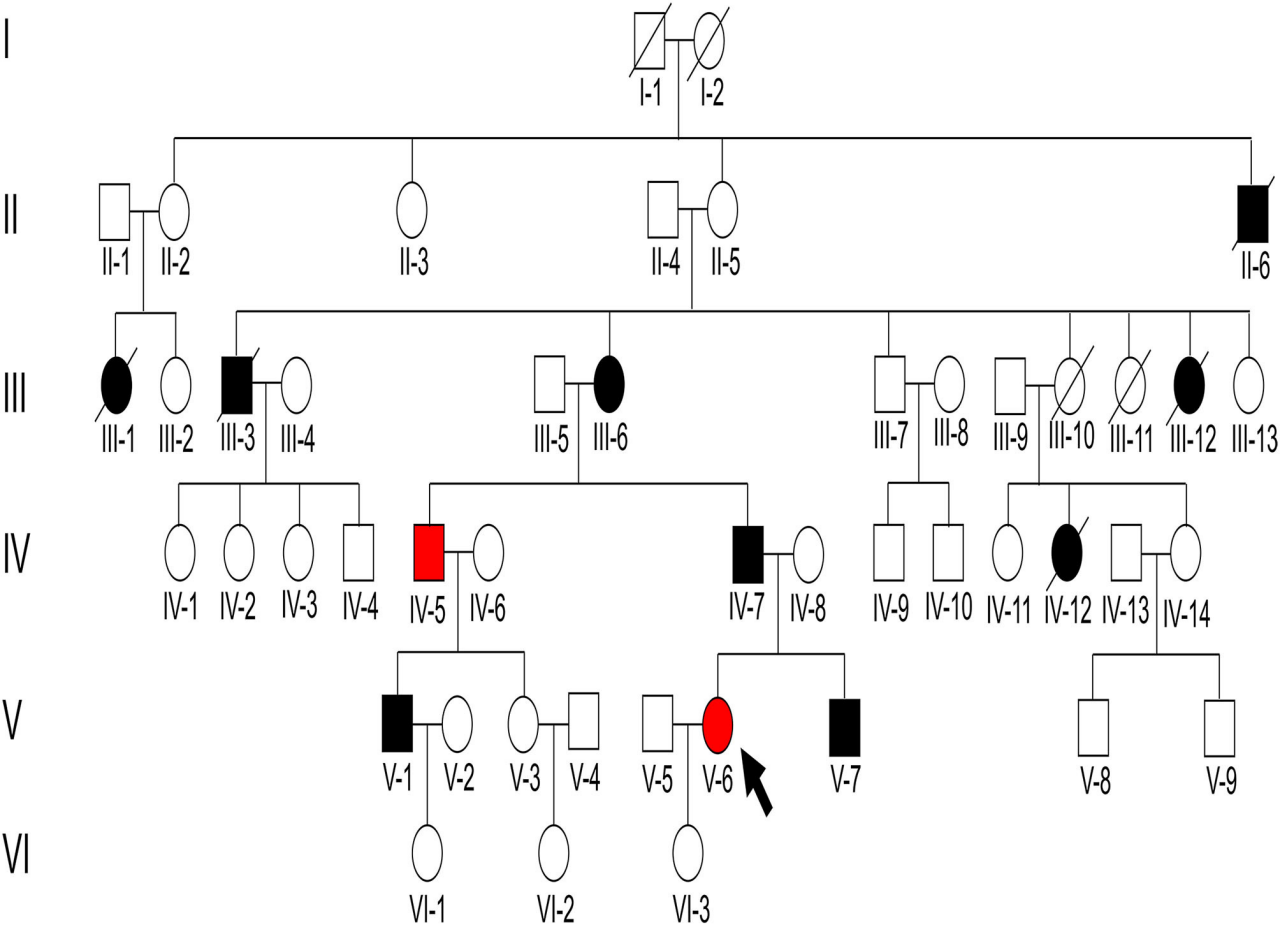
Pedigree structure of the family with immunoglobulin A (IgA) nephropathy (IgAN). There were 47 individuals in the Chinese six-generation IgA pedigree with no close relatives that were married. The arrow indicates the proband, black-filled shapes indicate abnormal proteinuria and/or hematuria results, red-filled shapes indicate patients diagnosed as IgAN, and unfilled shapes indicate unaffected individuals.

The proband was a 28-year-old Chinese woman (V-6) (Figure 1). She presented with proteinuria (++), hematuria, normal blood pressure, and normal renal function based on a pregnancy examination 4 years prior (at the age of 24 years). No renal biopsy or treatment was performed at that time. She was admitted to the hospital because her serum creatinine (Scr) level had increased to 200.5 μmol/l 1 week before admission.

**Figure 2:**
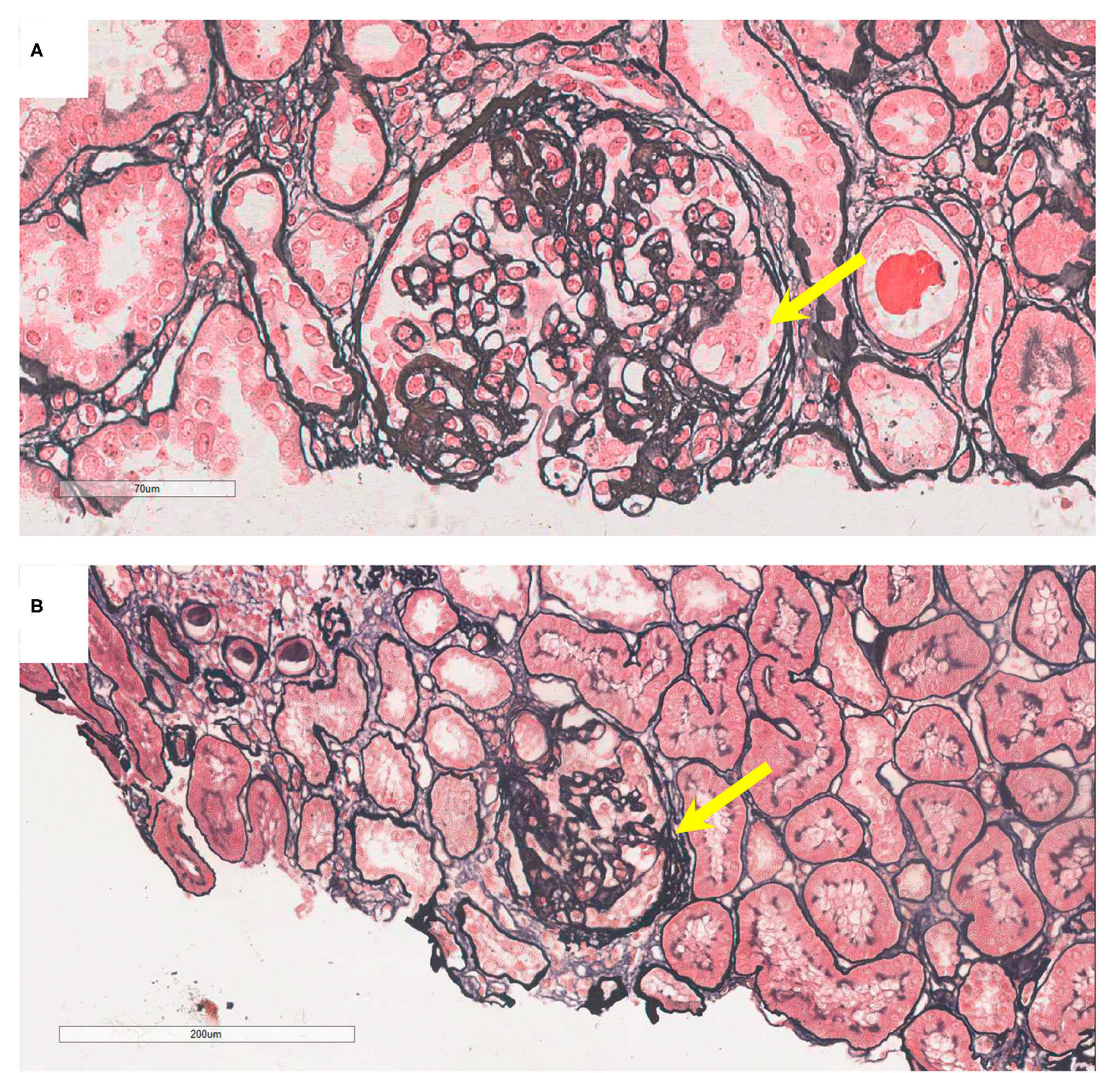
Pathology presentation of patients V-6 and IV-5. **(A)** Patient V-6: light microscopy showed segmental sclerosis and podocyte hypertrophy (yellow arrow), by periodic acid-silver methenamine (PASM) staining; original magnification: 200X. **(B)** Patient IV-5: light microscopy showed segmental adhesion and podocyte hypertrophy (yellow arrow; PASM staining, 100X).

On admission, urine examination showed proteinuria (2.55 g/24 h, normal range: 0–0.15 g), hematuria (720/μl, normal range: 0–10/μl), and elevated Scr levels (230.7 μmol/l, normal range: 53–97 μmol/l). The patient was diagnosed with IgAN through a renal biopsy. Immunofluorescence revealed IgG–, IgA+++, C1q+, C3c+++, and FRA–, and light microscopy revealed mesangial hypercellularity, segmental sclerosis, and podocyte hypertrophy (Figure 2A). Electron microscopy was not performed. Although the proband received antihypertension and corticosteroid therapy, she progressed to ESKD 4 years after renal biopsy and had to undergo hemodialysis.

The 52-year-old uncle (IV-5) of the proband developed proteinuria (1.33 g/d) and hematuria (18/μl) at the age of 32 years. He underwent renal biopsy at the age of 46 years and was diagnosed with IgAN. Immunofluorescence showed IgG–, IgA++, IgM–, C3c–, C1q–, and FRA–, and granular deposits of IgA in the mesangial areas. Light microscopy revealed mesangial hypercellularity, segmental adhesion, and podocyte hypertrophy (Figure 2B). Electron microscopy was not performed. He was administered a renin-angiotensin system inhibitor, and his renal function is normal at present.

The 24-year-old younger brother (V-7) of the proband presented with hematuria, proteinuria, normal blood pressure, and normal renal function at the age of 15. No renal biopsy or treatment was performed at that time. At present, his blood pressure has increased to 155/107 mm Hg and urine examination showed red blood cells at 10–20/HP and proteinuria (+++). His serum albumin levels were slightly decreased (36.4 g/l), Scr was within the normal range (902.4 μmol/l), and renal ultrasound showed bilateral renal atrophy. No renal biopsy was performed. He is being provided maintenance hemodialysis.

The father (IV-7), cousin (V-1), and grandmother (III-6) of the proband presented with proteinuria and/or hematuria at the ages of 30, 18, and 50, respectively. Secondary factors inducing renal disease were not found, and no renal biopsy was performed for these patients. The father of the proband progressed to ESKD and received dialysis at the age of 53. The cousin (V-1) and grandmother (III-6) of the proband received renin-angiotensin system blocker therapy and maintained normal blood pressure, stable proteinuria (<0.5 g/d), and normal renal function. In addition, five immediate family members (II-6, III-1, III-3, III-12, and IV-12) presented with proteinuria before death. Of these, III-3 died of a stroke and the others (II-6, III-1, III-12, and IV-12) died of unknown reasons.

## Genetic Analysis

The genetic analysis in this case study complied with the Declaration of Helsinki principles and was approved by the Peking University First Hospital ethics committees (2018-99). Informed consent was obtained from all participants. The genomic DNA of individuals was extracted from peripheral blood cells using the salting-out technique (8). Ten family members (III-5, III-6, IV-5, IV-6, IV-7, IV-8, V-1, V-3, V-6, and V-7), including the proband, were whole-exome sequenced by next-generation sequencing. To identify causal genetic changes, variants in 625 nephropathy-associated genes were selected for further analysis, according to a previous study (9). According to the American College of Medical Genetics and Genomics (ACMG) guidelines, benign variants (MAF > 0.05) and likely benign variants were filtered out (Supplementary Figure S1). Of the remaining variants, we explored a likely pathogenic variant in *WT1* and a pathogenic variant in *NPHS1* (Supplementary Table 2) for further analysis.

We identified a pathogenic missense variant in the *WT1* gene (NM_024426.6: exon9 c.1397C>T; p.Ser466Phe, dbSNP: rs1421664466) in seven immediate family members (III-6, IV-5, IV-7, V-1, V-6, V-7, and VI-1; Table 1), all of which exhibited proteinuria, except a 4-year-old member. This indicated that the variant in *WT1* (exon9: c.1397C>T; p.Ser466Phe) cosegregated with the proteinuria phenotype in this family. This missense variant was determined to be located in exon 9 and within the third zinc finger of the protein, which results in a substitution from serine to phenylalanine at residue 466. Our results suggest that this pathogenic missense variant of *WT1* is related to the development of IgAN in this family.

**Table 1:** Overview of genotypic and phenotypic data of related members in this pedigree.

**Individual**	**Gender**	**Age**	Age at onset^*^	**Renal function**	**Renal Biopsy^#^**	**Gene variants^&^**	**WES**
III-5	Male	72	NA	Normal	NA	NA	Yes
III-6	Female	73	50/proteinuria/ hematuria	Normal	NA	*WT1*: exon9: c.1397C>T;p.Ser466Phe	Yes
						*NPHS1*: exon27: c.3478C>T;p.Arg1160Ter	
III-7	Male	71	NA	Normal	NA	*NPHS1*: exon27: c.3478C>T;p.Arg1160Ter	No
III-8	Female	70	NA	Normal	NA	NA	No
IV-4	Male	42	NA	Normal	NA	NA	No
IV-5	Male	52	32/proteinuria	Normal	IgAN	*WT1*: exon9: c.1397C>T;p.Ser466Phe	Yes
						*NPHS1*: exon27: c.3478C>T;p.Arg1160Ter	
IV-6	Female	54	NA	Normal	NA	NA	Yes
IV-7	Male	50	30/proteinuria/hematuria	ESKD	NA	*WT1*: exon9: c.1397C>T;p.Ser466Phe	Yes
IV-8	Female	49	NA	Normal	NA	*WT1*: exon10: c.1433-10G>A	Yes
IV-9	Male	45	NA	Normal	NA	NA	No
IV-10	Male	42	NA	Normal	NA	NA	No
V-1	Male	31	18/proteinuria	Normal	NA	*WT1*: exon9: c.1397C>T;p.Ser466Phe	Yes
V-2	Female	30	NA	Normal	NA	NA	No
V-3	Male	29	NA	Normal	NA	*NPHS1*: exon27: c.3478C>T;p.Arg1160Ter	Yes
V-4	Female	27	NA	Normal	NA	NA	No
V-6	Female	28	24/proteinuria/hematuria	ESKD	IgAN	*WT1*: exon9: c.1397C>T;p.Ser466Phe	Yes
						*WT1*: exon10: c.1433-10G>A	
V-7	Male	24	15/proteinuria/hematuria	ESKD	NA	*WT1*: exon9: c.1397C>T;p.Ser466Phe	Yes
						*WT1*: exon10: c.1433-10G>A	
VI-1	Female	4	NA	Normal	NA	*WT1*: exon9: c.1397C>T;p.Ser466Phe	No
VI-2	Female	5	NA	Normal	NA	*NPHS1*: exon27: c.3478C>T;p.Arg1160Ter	No

*WES, whole-exome sequencing; WT1, Wilms tumor suppressor 1; NPHS1, nephrin; IgAN, IgA nephropathy; ESKD, end-stage kidney disease*.

* *NA means renal function was normal so far*.

# *NA means family members did not take renal biopsy*.

& *NA means no gene variant was detected*.

In addition, we identified a stop-gain pathogenic variant in *NPHS1* (NM_004646.4: exon27: c.3478C>T; p.Arg1160Ter, dbSNP: rs267606919) in five immediate family members (III-6, III-7, IV-5, V-3, and VI-2). Of them, III-6 and IV-5 showed proteinuria, whereas III-7, V-3, and VI-2 showed normal urinary protein excretion. The *NPHS1* variant did not cosegregate in the affected family members.

**Figure 3:**
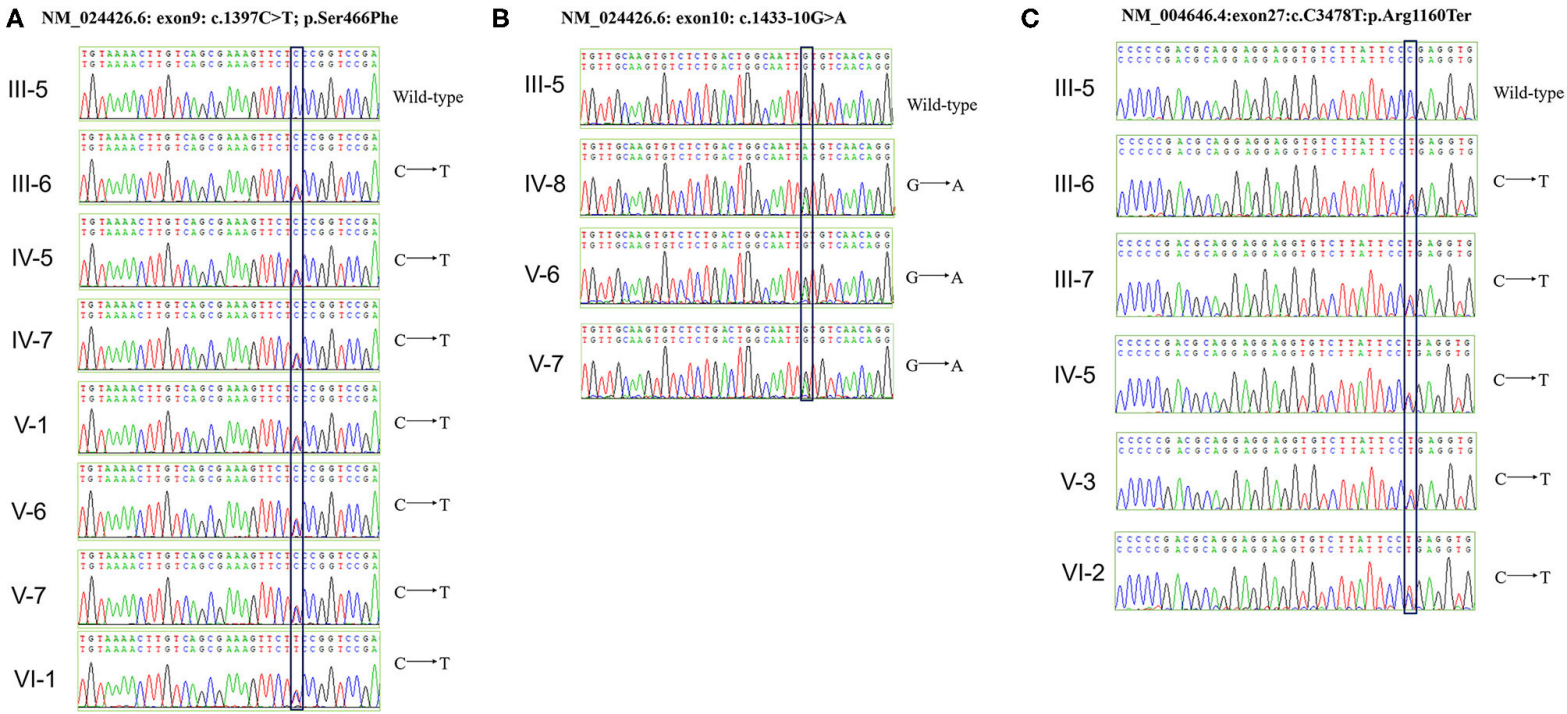
Verification of *WT1 and NPHS1* variants by Sanger sequencing. **(A)** The c.1397C>T; p.Ser466Phe mutation was verified in seven family members (III-6, IV-5, IV-7, IV-8, V-1, V-6, V-7, and VI-1). **(B)** The c.1433-10G>A mutation was verified in three family members (IV-8, V-6, and V-7). **(C)** The c.3478C>T; p.Arg1160Ter mutation was verified in five family members (III-6, III-7, IV-5, V-3, and VI-2).

Genetic testing also revealed that the proband, her younger brother, and his mother, carried another likely benign variant in the *WT1* gene (NM_024426.6: exon10: c.1433-10G>A), which cosegregated with the disease phenotype. As pathogenic variants of type IV collagen genes (*COL4A3/A4/A5*) have been recently reported to be causal factors for familial IgAN (10, 11), we checked for variants in these genes in the family but detected no pathogenic or likely pathogenic variant. Finally, all above mentioned three variants were verified by Sanger sequencing (Figure 3) and the primers used for sequencing were listed (Table S1).

## Discussion

Owing to the high and variable prevalence of genetic factors in different races and the familial aggregation of IgAN, their role in IgAN is widely accepted. To date, several genome-wide association studies in large sporadic IgAN populations have identified different genetic loci for IgAN susceptibility (10, 12). Whole-exome sequencing has proven to be a powerful tool for the identification of pathogenic mutations in familial diseases, especially Mendelian diseases. Therefore, using whole-exome sequencing, we identified a pathogenic missense variant in *WT1* that cosegregated with an abnormal proteinuria phenotype in a large IgAN pedigree of 47 members belonging to six generations.

In addition to hematuria, a considerable proportion of patients with IgAN exhibit proteinuria and podocyte lesions. An increased number of urinary podocytes and a decreased number of glomerular podocytes have been reported to be associated with IgAN prognosis (13, 14), suggesting the involvement of podocyte injury in IgAN. However, the exact mechanism of podocyte injury in IgAN remains unclear. Lai et al. found that when challenged with IgA deposition, mesangial cells become activated and produce cytokines that induce podocyte injury. The authors named this phenomenon mesangial-podocytic communication and proposed it as a pathogenic factor of podocyte injury in IgAN (15).

In this case study, we reported an IgAN pedigree with a pathogenic missense *WT1* gene variant that cosegregated with proteinuria. *WT1* is an important marker of normal podocytes in mature kidneys (16). The glomerular filtration barrier is composed of endothelial cells, glioblastoma (GBM), and podocytes. Podocyte injury is considered a crucial factor associated with proteinuria, a common phenotype in glomerular diseases (17). According to previous reports, *WT1* heterozygous mutations are associated with several kidney diseases with distinct podocyte lesions, such as FSGS, Frasier syndrome, and DDS, which are consistent with the presence of a missense variant in exon 8 or 9, encoding zinc finger proteins (6, 18, 19).

In our reported pedigree, the occurrence of proteinuria was seen in all members carrying the identified pathogenic missense variant in *WT1* (exon9: c.1397C>T; p.Ser466Phe). This variant has previously been identified in a 46-year-old female with isolated nephrotic proteinuria, and in her father who presented with chronic renal failure (20). In the adult kidney, *WT1* expression is limited to the podocytes and plays a crucial role in normal renal podocyte function (21). Mutations in *WT1* can induce dedifferentiation, abnormal proliferation, and morphological alterations in podocytes by affecting its zinc finger domain and disturbing the alternative splicing of ±KTS isoforms (22). The pathogenic variant in exon9, c.1397C>T; p.Ser466Phe, is located in the zinc finger 3 domain of the WT1 protein, and the Ser > Phe change might induce a structural change in the third zinc finger (20). Sakamoto et al. reported that PKA phosphorylation of Ser-393 could decrease the transcriptional activity of *WT1* by repressing its DNA-binding ability (23). Recently, Nagano et al. studied the transcriptional activity of *WT1* through a systematic review and reported that mutations in both the DNA-binding site and C2H2 zinc finger structure may cause severe clinical phenotypes (24). Based on the aforementioned evidence, we postulated that this pathogenic missense variant in *WT1* is the causal factor that induced proteinuria in the IgAN pedigree analyzed in this case study.

Furthermore, we also identified a pathogenic stop-gain variant of *NPHS1* (exon27: c.3478C>T; p.Arg1160Ter), which changes the length of the protein nephrin by causing premature termination, in this family. This stop-gain variant has previously been reported in a patient with congenital nephrotic syndrome of the Finnish type (25). Nephrin plays an important role in the organization of the slit diaphragm (26). Homozygous or compound heterozygous *NPHS1* mutations have been observed in patients with congenital nephrotic syndrome, late-onset steroid-resistant nephrotic syndrome, and FSGS (27–29). In this family, five members (III-6, III-7, IV-5, V-3, and VI-2) were heterozygous for this variant and only two—the grandmother of the proband with proteinuria (III-6) and the uncle of the proband diagnosed with IgAN (IV-5)—had a kidney-associated phenotype. The other three members heterozygous for the *NPHS1* variant (III-7, V-3, and VI-2) showed no kidney-associated phenotype, indicating that this variant did not cosegregate with the disease. Although the grandmother and uncle of the proband carried both *WT1* and *NPHS1* missense variants, until the time this case study was conducted, their renal functions were normal, the clinical phenotypes were mild, and disease progression was slow.

In addition, we also found that two individuals in this pedigree with severe kidney phenotypes—the proband and her younger brother—carried another *WT1* variant (exon10: c.1433-10G>A), which they inherited from their mother, other than the missense variant (exon9: c.1397C>T; p.Ser466Phe), which they inherited from their father. Although the c.1433-10G>A variant was not predicted to be a splicing variant by splicing algorithms (BDGP and ASSP) and was therefore regarded as benign based on the ACMG guidelines, it cosegregated with the disease severity phenotype. The proband and her younger brother progressed to ESKD at ages 28 and 24 years, respectively. In contrast, other individuals in the pedigree exhibiting only the missense *WT1* variant presented with mild kidney injury and slow disease progression. Therefore, we speculate that the c.1433-10G>A variant may also contribute to disease progression and the development of the clinical phenotype in this family and suggest that its influence on *WT1* must be investigated further.

This case study is limited in that not all the family members with kidney disease were biopsied and, therefore, we could not confirm their diagnosis of having developed IgAN. Moreover, renal biopsy samples were not evaluated by electron microscopy, and we failed to accurately evaluate podocyte and GBM lesions. In summary, here, we report an IgAN pedigree with a pathogenic missense heterozygous *WT1* variant (c.1397C>T; p.Ser466Phe) and suggest that *WT1* pathogenic missense variant-induced primary podocyte injury is responsible for the proteinuria phenotype and IgAN progression in this pedigree.

## Ethics Statement

The studies involving human participants were reviewed and approved by The Medical Ethics Committee of Peking University First Hospital. Written informed consent to participate in this study was provided by the participants' legal guardian/next of kin.

## Author Contributions

Research idea and study design by LZ and SS. Data acquisition and Data analysis/interpretation by QL. Supervision or mentorship by LZ, SS, DX, JL, and HZ. Each author contributed important intellectual content during manuscript drafting or revision and accepts accountability for the overall work by ensuring that questions pertaining to the accuracy or integrity of any portion of the work are appropriately investigated and resolved. All authors contributed to the article and approved the submitted version.

## Funding

This case study was supported by grants from the National Key Research and Development Program of China (2020YFC2005003), the National Science Foundation of China (81922013, 81970598, and 82070733), the National Science Foundation of Beijing (7192209 and 7202206), the Youth Development Project from Peking University Health Science Center (BMU2021PY004), and the CAMS Innovation Fund for Medical Sciences (2019-I2M-5-046).

## Conflict of Interest

The authors declare that the research was conducted in the absence of any commercial or financial relationships that could be construed as a potential conflict of interest.

## Publisher's Note

All claims expressed in this article are solely those of the authors and do not necessarily represent those of their affiliated organizations, or those of the publisher, the editors and the reviewers. Any product that may be evaluated in this article, or claim that may be made by its manufacturer, is not guaranteed or endorsed by the publisher.
