# Supplementary material for: Case Report: A Pathogenic Missense Variant of WT1 Cosegregates With Proteinuria in a Six-Generation Chinese Family With IgA Nephropathy
Source: Front Med (Lausanne). 2022 Jan 31;8:810940. doi: 10.3389/fmed.2021.810940 (PMC8841721; doi:10.3389/fmed.2021.810940)

**Table S1. Primers used for sequencing of identified variants.**

| **Gene** | **Variants** | **Primer sequence (5’-3’)** | |
| --- | --- | --- | --- |
|  |  | **Forward** | **Reverse** |
| *WT1* | exon9: c.1397C>T; p.Ser466Phe | GCAGATGCAGACATTGCAG | TCCAATCCCTCTCATCACAA |
| *NPHS1* | exon27: c.3478C>T; p.Arg1160Ter | GCCGTTAGCATCAGGTTGA | GTTGGAGTGGAAGGGAGACA |
| *WT1* | exon10: c.1433-10G>A | AAGGCTGCCTGGGAAATG | GAGGGAGACCCCTCAAAGC |

*WT-1*: Wilms tumor 1; *NPHS1*: Nephrin.

**Table S2. Candidate contributing variants identified in the family.**

| **Position** | **Gene** | **Variants** | **Variant interpretation** |
| --- | --- | --- | --- |
| Chr11: 32392022 | *WT1* | exon9:c.1397C>T; p.Ser466Phe | Likely Pathogenic |
| Chr19: 35831056 | *NPHS1* | exon27:c.3478C>T; p.Arg1160Ter | Pathogenic |
| Chr11: 32389189 | *WT1* | exon10:c.1433-10G>A | Likely Benign |

**Figure Legend**

**Figure S1. Filtering process of the variants identified by whole exome sequencing**

**in this pedigree.** MAF: minor allele frequency; LP: Likely Pathogenic; P: Pathogenic;

* Guruswamy Sangameswaran KD, Baradhi KM. Focal Segmental Glomerulosclerosis. [Updated 2021 Jul 26]. In: StatPearls [Internet]. Treasure Island (FL): StatPearls Publishing.

**Figure S1. Filtering process of the variants resulting from whole exome sequencing.**


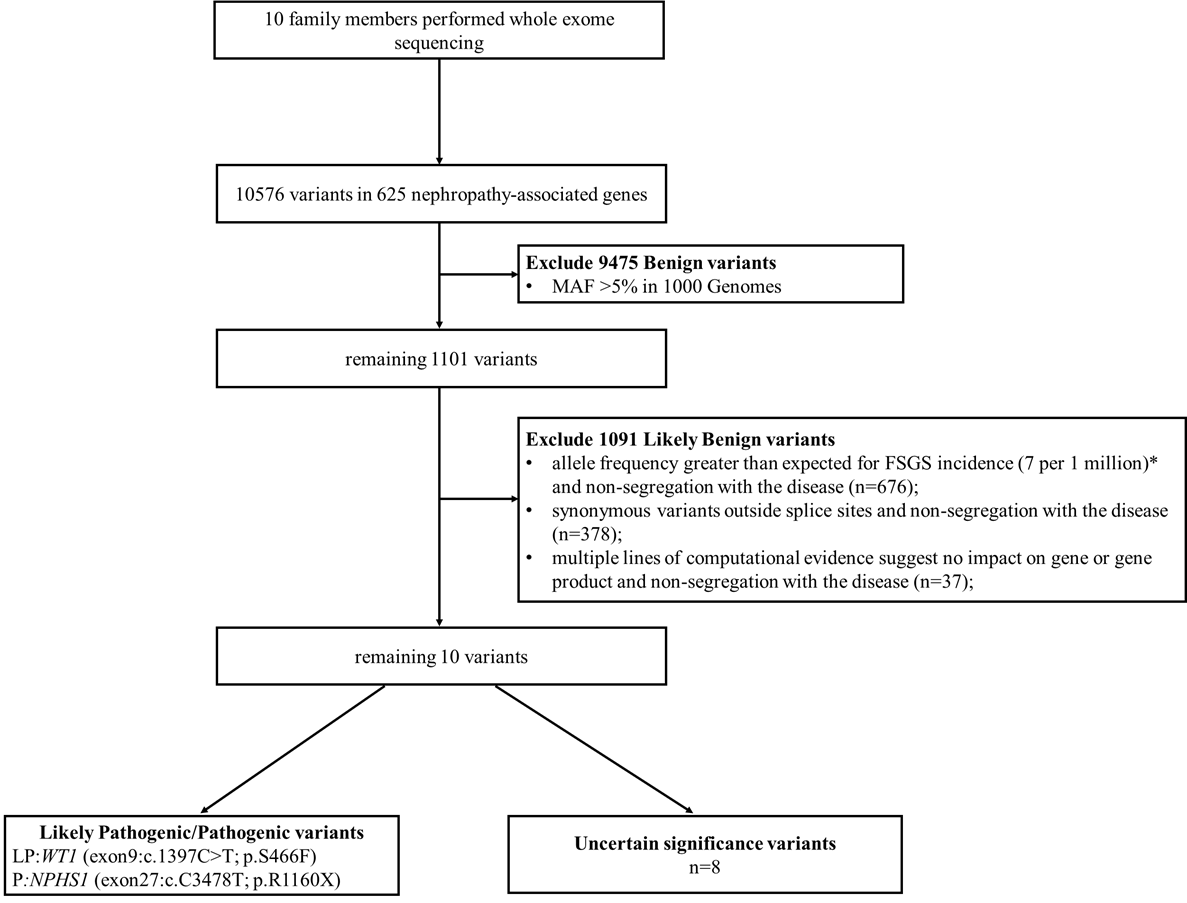

Supplement: Supplementary file 1 [file Data_Sheet_2.docx]
